# Microbial Communities and Functions in the Rhizosphere of Disease-Resistant and Susceptible *Camellia* spp.

**DOI:** 10.3389/fmicb.2021.732905

**Published:** 2021-10-18

**Authors:** Jun Li, Chenhui Zhang, Xinjing Qu, Ziqiong Luo, Sheng Lu, Yakov Kuzyakov, Hattan A. Alharbi, Jun Yuan, Genhua Niu

**Affiliations:** ^1^Key Laboratory of Cultivation and Protection for Non-Wood Forest Trees, Ministry of Education, Central South University of Forestry and Technology, Changsha, China; ^2^Department of Agricultural Soil Science, Department of Soil Science of Temperate Ecosystems, Georg-August-Universität Göttingen, Göttingen, Germany; ^3^Agro-Technological Institute, RUDN University, Moscow, Russia; ^4^Institute of Environmental Sciences, Kazan Federal University, Kazan, Russia; ^5^College of Food and Agriculture Sciences, King Saud University, Riyadh, Saudi Arabia; ^6^Texas A&M AgriLife Research and Extension Center at Dallas, Texas A&M University, Dallas, TX, United States

**Keywords:** *Camellia yuhsienensis*, *Camellia oleifera*, rhizosphere microbiome and functions, plant growth promoting microorganisms, soilborne phytopathogens

## Abstract

Oil tea (*Camellia* spp.) is endemic to the hilly regions in the subtropics. *Camellia yuhsienensis* is resistant to diseases such as anthracnose and root rot, while *Camellia oleifera* is a high-yield species but susceptible to these diseases. We hypothesize that differences in the rhizosphere microbial communities and functions will elucidate the resistance mechanisms of these species. We used high-throughput sequencing over four seasons to characterize the rhizosphere microbiome of *C. oleifera* (Rhizo-Sus) and *C. yuhsienensis* (Rhizo-Res) and of the bulk soil control (BulkS). In Rhizo-Res, bacterial richness and diversity (Shannon index) in autumn and winter were both higher than that in Rhizo-Sus. In Rhizo-Res, fungal richness in autumn and winter and diversity in summer, autumn, and winter were higher than that in Rhizo-Sus. The seasonal variations in bacterial community structure were different, while that of fungal community structure were similar between Rhizo-Res and Rhizo-Sus. Gram-positive, facultatively anaerobic, and stress-tolerant bacteria were the dominant groups in Rhizo-Sus, while Gram-negative bacteria were the dominant group in Rhizo-Res. The significant differences in bacterial and fungal functions between Rhizo-Sus and Rhizo-Res were as follows: (1) in Rhizo-Sus, there were three bacterial and four fungal groups with plant growth promoting potentials, such as *Brevibacterium epidermidis* and *Oidiodendron maius*, and one bacterium and three fungi with pathogenic potentials, such as *Gryllotalpicola* sp. and *Cyphellophora sessilis*; (2) in Rhizo-Res, there were also three bacteria and four fungal groups with plant-growth-promoting potentials (e.g., *Acinetobacter lwoffii* and *Cenococcum geophilum*) but only one phytopathogen (*Schizophyllum commune*). In summary, the rhizosphere microbiome of disease-resistant *C. yuhsienensis* is characterized by a higher richness and diversity of microbial communities, more symbiotic fungal communities, and fewer pathogens compared to the rhizosphere of high-yield but disease-susceptible *C. oleifera.*

## Introduction

The rhizosphere, where the soil volume is directly affected by roots, is a narrow zone with high abundance of microorganisms and is one of the most complex ecosystems on Earth (Kuzyakov and Razavi, 2019; Vetterlein et al., 2020). The function of many microbial groups is to facilitate plant growth and rhizosphere health through their functions. Four important beneficial microbial groups are commonly found in the rhizosphere: (i) nitrogen-fixing bacteria, (ii) mycorrhizal fungi, (iii) plant-growth-promoting rhizobacteria (PGPR), and (iv) biocontrol microorganisms, with the ability to protect plant roots against pathogens through excretion of metabolites or via competition for niche (Mendes et al., 2013). Deleterious microorganisms can invade root tissues, cause diseases or retard root growth, and reduce yield through the excretion of toxic metabolites (Kremer, 2006). Both beneficial and deleterious microorganisms coexist in the rhizosphere. For example, *Bacillus megaterium* is present in the rhizosphere of *Camellia sinensis* and has the ability to promote plant growth by solubilizing phosphate, producing indole acetic acid (IAA) and siderophores, and protecting plants against brown root rot disease, caused by rhizosphere fungus *Fomes lamaoensis* (Chakraborty et al., 2006; Huang et al., 2019).

Rhizosphere microorganisms are affected directly by the host plant and indirectly by seasonal rainfall, temperature, and light intensity, as these factors modify the rhizodeposition (Kuzyakov and Cheng, 2001; Taketani et al., 2016; Zhang X. et al., 2020). Plant diseases caused by microorganisms are often associated with extreme drought or rainfall (Meisner and Boer, 2018). Pathogens can tolerate the negative effects of drought better than plants and infect the stressed plants through synthesis of toxins and cell-wall-degrading enzymes (Dikilitas et al., 2016). Most bacteria that are better adapted to droughts and other resource-limiting conditions are Gram-positive and are K-strategists or oligotrophs. In contrast, Gram-negative bacteria (mainly *r*-strategists) have an advantage under nutrient-rich conditions in wet soil (Chen et al., 2016).

Oil tea is an indispensable woody oilseed plant that is endemic to the hilly areas in subtropical climate (Liu et al., 2017), growing on soils with low pH, low organic matter, and limited phosphorous content (Li et al., 2019). Low yields of *Camellia*, caused by barren ground, nutrient shortage, and plant diseases and pests, have been the predominant limiting factor for the development of the oil tea industry for many years. After years of breeding, some nutrient-efficient and disease-resistant species or cultivars have been selected. *Camellia oleifera* “Huashuo” is a widely planted oil tea species with high yield (Tan et al., 2011), but it is disease sensitive and susceptible to anthracnose, root knot nematode, and root rot diseases (Wang et al., 2017; Xu et al., 2020; Zhu et al., 2020). *Camellia yuhsienensis*, another important oil tea species, is resistant to these diseases (Yang et al., 2004; Wei et al., 2013). According to previous studies, the yield of *C. oleifera* is much higher than *C. yuhsienensis* (Zou, 2010; Tan et al., 2011), while the disease resistance of *C. yuhsienensis* is much better than *C. oleifera* (Supplementary Table 1; Yang et al., 2004; Jin et al., 2009; Chen et al., 2021). Nutrient absorption from the soil and plant disease resistance is also affected by the soil microbiome, especially the rhizosphere microorganisms (Bob et al., 1987; Jansson and Hofmockel, 2020). Although the rhizosphere microbial communities have been investigated in *C. oleifera* (Zhang P. et al., 2020) and *C. yuhsienensis* (Li et al., 2020), it remains difficult to compare differences in rhizosphere microbial community structures and functions due to inconsistent climate, soil conditions, and management practices. More importantly, none of the previous studies have reported on the microbial functions and their role in oil tea disease susceptibility and growth.

Because of the self-pollination incompatibility in oil tea species, multiple cultivars are planted in the same orchard (Zhou et al., 2020). Plants are able to shape or modify their rhizosphere microbiome through the excretion of specific secondary metabolites, even when grown in one planation (Berendsen et al., 2012). Therefore, we hypothesized that, in one planation (same climate, soil type, and management), *C. oleifera* and *C. yuhsienensis* each developed unique rhizosphere microbial communities, and the differences in rhizosphere microbial diversity, structure, and functions explain the different resistances to disease in these two *Camellia* species. High-throughput sequencing of taxonomic composition and functions of the rhizosphere microbiome were used to verify our hypotheses.

## Materials and Methods

### Site Description

The sampling site was in Wangcheng district, Changsha City, Hunan, China (N 28°30′, E 113°20′). To study the effect of mixed planation on growth, health, and production of oil tea, two 1-year-old *Camellia* oil species, namely, *C. yuhsienensis* and *C. oleifera* “Huashuo,” were planted in 2011 at the sampling site at a 1:1 ratio. Plant spacing was 2 m within rows, and row spacing was 3 m. The climate is a subtropical monsoon with mean annual rainfall and temperature of 1,370 mm and 17°C, respectively. The soil at the experimental site is a Quaternary red clay (classified as Lixisols in the World Reference Base for Soil Resources) with a pH of 4.3. Total organic carbon content (TOC) was 11.5 ± 0.6 g⋅kg^–1^, and total nitrogen (TN) content was 870 ± 25 mg⋅kg^–1^. Available phosphorus (AP) content was 4.9 ± 1.7 mg⋅kg^–1^ and available potassium (AK) content was 134 ± 11 mg⋅kg^–1^ (Supplementary Table 2). The understory contains some wild weeds, which were mowed twice a year.

### Experimental Design and Sampling

Growth characteristics of *C. oleifera* and *C. yuhsienensis* were collected 7 years after planting (Supplementary Table 1). Soil samples were collected at the fruit enlargement stage (October 23, 2018, autumn, Au), dormancy stage (January 19, 2019, winter, Wi), spring shoot growth stage (April 5, 2019, spring, Sp), and flower bud rapid growth stage (July 22, 2019, summer, Su). Three separate plots of 20 m × 20 m were selected in the mixed plantation. Five healthy trees of each species were selected using an “S” type design from the center of each quadrat. Four points in each quadrat were selected at 0.5–1 m distance from the trunk, and samples of the soil rhizosphere of *C. oleifera* (Rhizo-Sus) and *C. yuhsienensis* (Rhizo-Res) were collected as described by Koranda et al. (2011). Samples of a corresponding bulk soil (BulkS) at 0–20 cm depth were also collected as a reference control in the middle of the rows, approximately 1.5 m distance from the trunk. Subsamples of the rhizosphere samples or the bulk soil were mixed well and combined into one sample. Soil samples were stored in dry ice and transported back to the laboratory for further processing. After removal of debris and roots, samples were mixed well, ground, and ran through a sieve (<2 mm). One portion of each soil sample was used to determine the physicochemical properties after air-drying in the shade, while the remainder portion was stored at −80°C for high-throughput sequencing at Genedenovo Biotechnology Co., Ltd. (Guangzhou, China).

The soil physicochemical properties were measured according to Li et al. (2020). Soil temperature at 0–20 cm depth was measured using a Wdsen Electronic temperature recorder (iButton DS1925) during the entire experimental period (Supplementary Figure 1).

### Bacterial and Fungal Community Assessment

#### DNA Extraction and PCR Amplification

A soil sample of 2–3 g dry weight was used for microbial DNA extraction using HiPure Soil DNA Kits (Magen, Guangzhou, China) according to manufacturer’s protocol. The 16S rDNA V3–V4 region of the ribosomal RNA gene was amplified by PCR using primer 341F, 5′-CCTACGGGNGGCWGCAG-3′, and 806R, 5′-GGACTACHVGGGTATCTAAT-3′. The PCR amplification of 16S rDNA was conducted as described by Li et al. (2018). The internal transcribed spacer (ITS) rDNA region of the ribosomal RNA gene was amplified by PCR using primers ITS3-KYO2 (F), 5′-GATGAAGAACGYAGYAA-3′, and ITS4 (R), 5′-TCCTCCGCTTATTGATATGC-3′ (Toju et al., 2012). The ITS region of the eukaryotic ribosomal RNA gene was amplified by PCR (95°C for 2 min, followed by 27 cycles at 98°C for 10 s, 62°C for 30 s, and 68°C for 30 s, and a final extension at 68°C for 10 min). The PCR were performed in triplicate using 50-μl mixtures containing 5 μl of 10 × KOD buffer, 5 μl of 2 mM dNTPs, 3 μl of 25 mM MgSO_4_, 1.5 μl of each primer (10 μM), 1 μl of KOD polymerase, and 100 ng of template DNA.

#### Illumina Novaseq6000 Sequencing

Amplicons were extracted from 2% agarose gels and purified using an AxyPrep DNA Gel Extraction Kit (Axygen Biosciences, Union City, CA, United States) according to the manufacturer’s instructions. The qualified amplicon mixture was then sequenced on the Illumina Novaseq6000 platform to generate 2 × 250 bp paired-end reads. The raw reads were deposited into the National Center for Biotechnology Information (NCBI) Sequence Read Archive (SRA) database (accession number PRJNA742848). The link of this BioProject is https://dataview.ncbi.nlm.nih.gov/object/PRJNA742848?reviewer=haouuog1lqfrksad66m99e11p5.

### Statistical and Bioinformatics Analysis

Raw reads were further filtered using FASTP by removing reads containing more than 10% unknown nucleotides (N) and reads with < 50% of bases with quality scores (*Q*-value) > 20 (Chen et al., 2018). Paired-end clean reads were merged as raw tags using FLASH (version 1.2.11) with a minimum overlap of 10 bp and mismatch error rates of 2% (Magoè and Salzberg, 2011). The effective tags were clustered into operational taxonomic units (OTUs) of ≥ 97% similarity using the UPARSE (version 9.2.64) pipeline (Edgar, 2013). The tag sequence with the highest abundance was selected as the representative sequence within each cluster. The tags and OTUs of the bacteria and fungi are presented in Supplementary Tables 3, 4. To ensure the reproducibility and validity of the microbial data, the data extraction flat and dilution curves were processed before analysis (Liang et al., 2017). The dilution curve of the Sobs index indicated the presence of more bacteria and fungi if sequencing was continued, but the dilution curve plateau of the Shannon’s index was reached early, indicating that the number of reads was sufficient for this research (Supplementary Figure 2).

Venn analysis, used to identify unique and common OTUs and species among different compartments, was performed using the R “VennDiagram” package (version 1.6.16) (Chen and Boutros, 2011). The OTU rarefaction curves and rank abundance curves were plotted using the R “ggplot2” package (version 2.2.1) (Wickham, 2006). Alpha diversities of bacteria and fungi (Sobs, Shannon, and Chao 1 index) were calculated in QIIME (version 1.9.1) (Caporaso et al., 2010), and differences in the alpha diversities of bacteria and fungi among treatments were calculated using one-way analysis of variance (ANOVA). Principal coordinates analysis (PCoA), permutational multivariate analysis of variance (MANOVA) (Permanova), and cluster dendrogram based on unweighted uniFrac distances were used to evaluate the influence of plant roots and seasonal dynamics on the bacterial and fungal community structures. The unweight uniFrac distance matrix and Permanova was calculated in R project GuniFrac package (version 1.0) and Vegan package (version 2.5.3), respectively. Ape and Vegan packages were used for PCoA analysis, and ggplot2 package was used for visualization in R. A least discriminant analysis (LDA) effect size (LEfSe) taxonomic cladogram was used to identify specific (LDA > 3.0) bacteria and fungi (LDA > 3.0) in the treatments using LEfSe software (Segata et al., 2011). Ternary plots detected by Kruskal–Wallis H analysis were used to determine the specific species among Rhizo-Sus, Rhizo-Res, and BulkS during the entire year using the R “labdsv” package (version 2.0-1) and R “ggplot” (Roberts, 2016). The Kyoto Encyclopedia of Genes and Genomes (KEGG) pathway analysis of the OTUs for prediction of bacterial functions was inferred using Tax4Fun (version 1.0) (Aßhauer et al., 2015). Microbiome phenotypes of bacteria were classified using BugBase (Ward et al., 2017). The functional group (Guild) of the fungi (relative abundance > 0.01% in the rhizosphere) was inferred using FUNGuild (version 1.0) (Nguyen et al., 2016). The bacterial functions and the fungal trophic modes among Rhizo-Sus, Rhizo-Res, and BulkS were calculated by Wilcoxon rank sum test. The fungal functional groups (guild) among Rhizo-Sus, Rhizo-Res, and BulkS were calculated using the Kruskal–Wallis *H* test.

Among treatments were calculated using one-way analysis of variance was used to determine the differences in soil physicochemical properties among Rhizo-Sus, Rhizo-Res, and BulkS during the entire year. Pearson’s correlation coefficients between soil physicochemical properties and alpha diversities were calculated by Omicshare tools, a free online platform for data analysis (Denovo, 2021).

## Results

### Soil Microbial Community Structure

#### Microbial Composition and Diversity

Regardless of whether the soil sampled was bulk or rhizosphere, the dominant bacteria phyla were Chloroflexi (14–43% of total bacteria sequencing), Acidobacteria (8–35%), Proteobacteria (12–28%), Actinobacteria (9–29%), and Planctomycetes (5–13%) (Supplementary Figure 3A). The dominant fungi were Ascomycota (61–96% of total fungi sequencing) and Basidiomycota (2–34%) (Supplementary Figure 3B).

**FIGURE 1:**
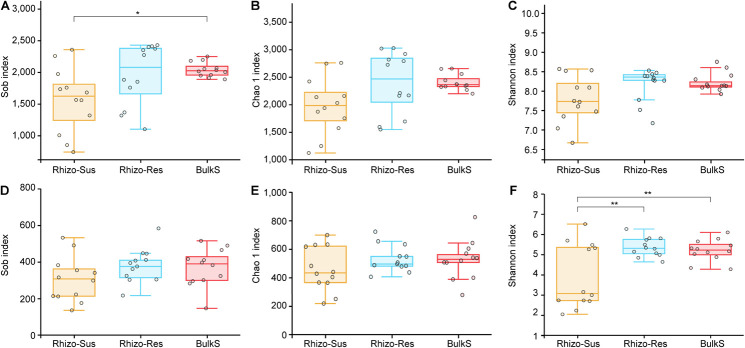
Box plots of bacterial **(A–C)** and fungal **(D–F)** Sobs and Chao 1 and Shannon index in disease susceptible *C. oleifera* rhizosphere (Rhizo-Sus, yellow), disease resistant *C. yuhsienensis* rhizosphere (Rhizo-Res, blue), and bulk soil (BulkS, red). The line in the box indicates the median of each compartment. The line over and below the box indicates the maximum and minimum value, respectively. The top and bottom line of the box indicates the upper and lower quartile, respectively. The circles indicate each sample. The * and ** over the boxes indicate the significant differences based on Tukey’s HSD test among compartments at *p* < 0.05 and *p* < 0.01, respectively.

Bacterial richness and diversity were higher in Rhizo-Res than in Rhizo-Sus in autumn and winter (*p* < 0.05, Supplementary Table 5). Fungal diversity was higher in Rhizo-Res than in Rhizo-Sus in summer and autumn (*p* < 0.05). Over a 1-year period, the fungal diversity in Rhizo-Res and BulkS was higher than in Rhizo-Sus (*p* < 0.05) (Figure 1F).

**FIGURE 2:**
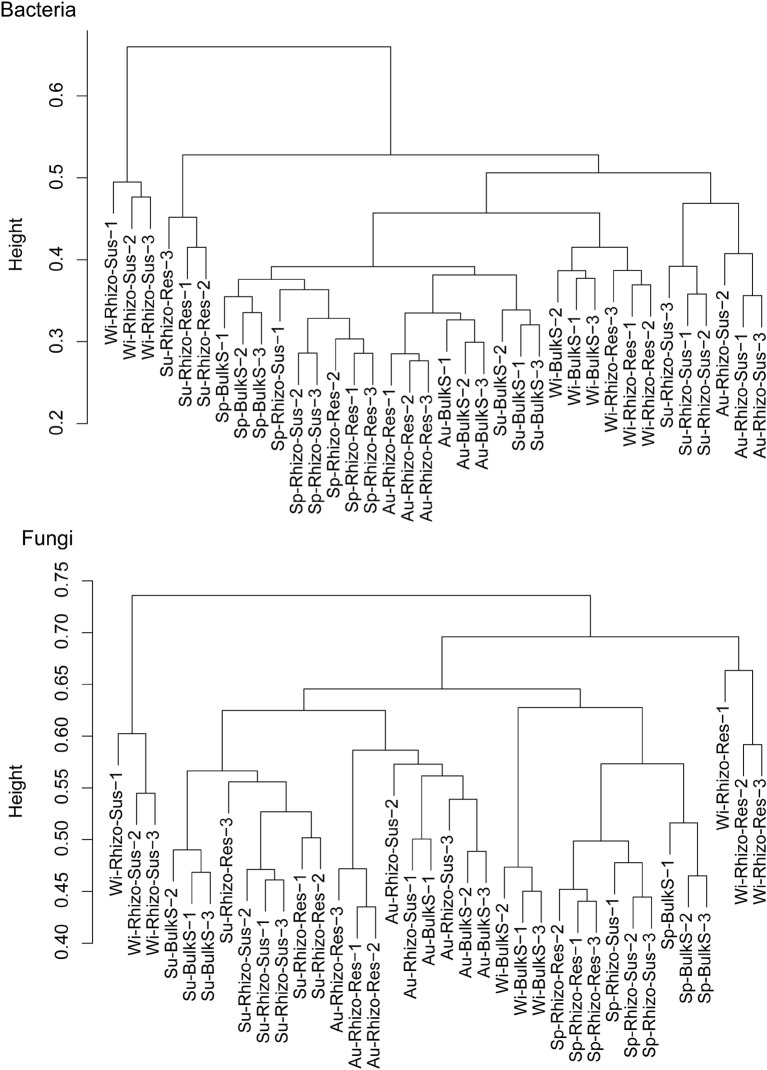
Cluster dendrogram of bacterial and fungal communities based on unweighted uniFrac distance. Sp, Su, Au, and Wi indicates spring, summer, autumn, and winter, respectively. Rhizo-Sus, Rhizo-Res, and BulkS indicate disease-susceptible *C. oleifera* rhizosphere, disease-resistant *C. yuhsienensis* rhizosphere, and bulk soil, respectively, *N* = 3. Height in cluster dendrogram indicates the distance of microbial communities among each sample.

Both season and *Camellia* species had a marked influence on bacterial and fungal community structure (*p* < 0.05) (Figure 2 and Supplementary Figure 4). According to the cluster dendrogram (Figure 2), the bacterial community structure in Rhizo-Sus was different from Rhizo-Res and BulkS in winter. In summer, the bacterial community in Rhizo-Res was different from Rhizo-Sus and BulkS. The fungal community structures were similar among Rhizo-Sus, Rhizo-Res, and BulkS during the year, except in winter (Supplementary Figure 4). Over the whole year, the trend of bacterial community development in Rhizo-Res was similar to BulkS (along with axis PCO2) but different from that in Rhizo-Sus (along with axis PCO1). The trend of fungal community structure in Rhizo-Res was similar to Rhizo-Sus (along with axis PCO1, Supplementary Figure 4).

#### Specific Microbial Species in the Rhizosphere of Oil Tea Plants

**FIGURE 3:**
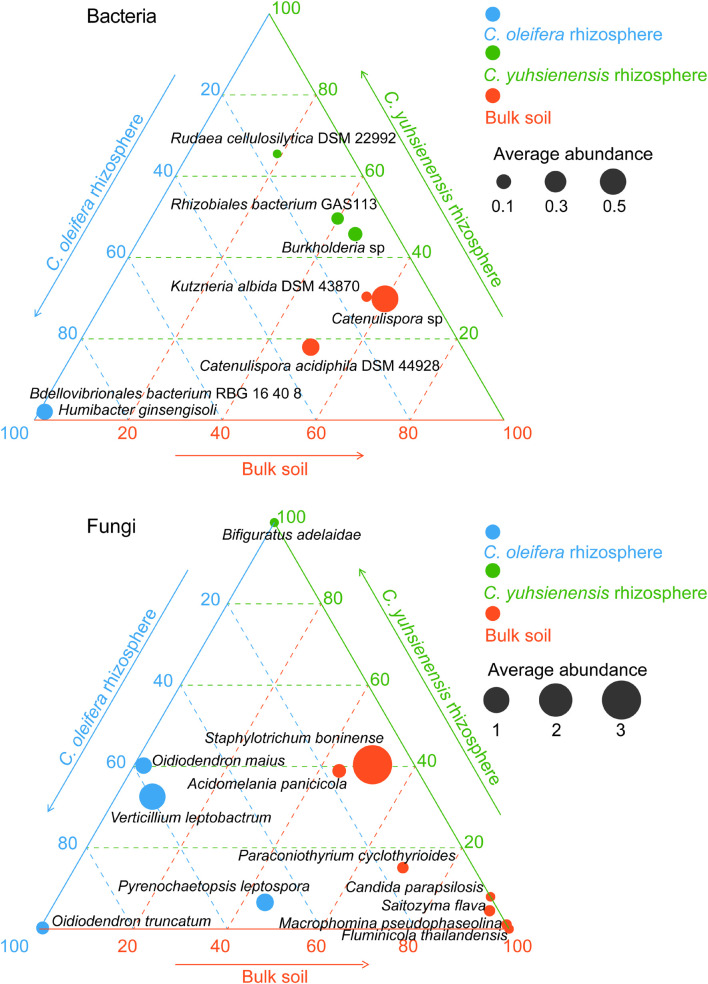
Distribution of specific bacterial and fungal species across disease-susceptible *C. oleifera* rhizosphere (blue), disease-resistant *C. yuhsienensis* rhizosphere (green), and bulk soil (red) in ternary plots. The circle size and position indicates the average relative abundance (%) and the ratio of relative abundance of each microbial group with *C. oleifera* rhizosphere, *C. yuhsienensis* rhizosphere, and bulk soil (conducted using Kruskal–Wallis analysis, *p* < 0.05), respectively, *N* = 12.

**FIGURE 4:**
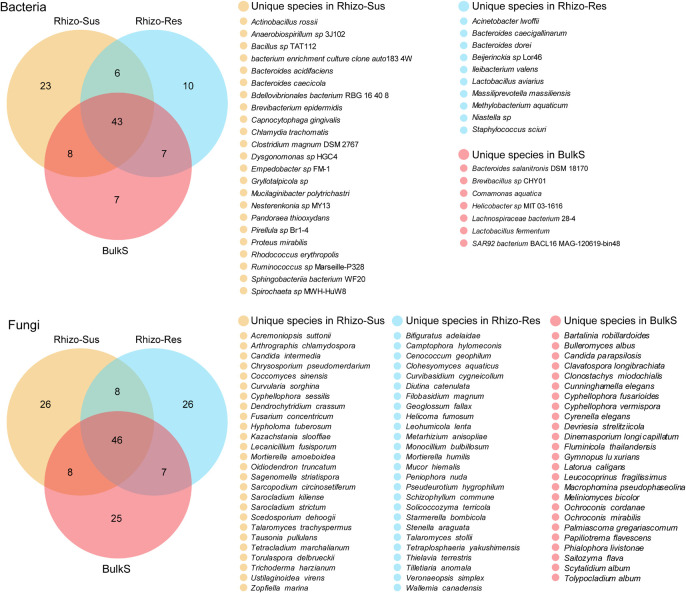
Venn diagram of bacteria and fungi at species level among *C. oleifera* rhizosphere (Rhizo-Sus), *C. yuhsienensis* rhizosphere (Rhizo-Res), and bulk soil (BulkS), *N* = 12. Yellow, blue, and red color indicate the unique species that are only presented in Rhizo-Sus, Rhizo-Res, or BulkS, respectively. The cross-color block indicates the shared species between Rhizo-Sus and Rhizo-Res, or Rhizo-Sus and BulkS, or Rhizo-Res and BulkS, or among Rhizo-Sus, Rhizo-Res, and BulkS. The quantity of unique and shared species was written in each color block of Venn diagram, respectively.

Species with higher relative abundance in one rhizosphere or bulk soil (*p* < 0.05) (Figure 3) or species unique to Rhizo-Sus, Rhizo-Res, and BulkS (Figure 4) are defined as specific microorganisms. According to Ternary plots (Figure 3), the specific species across the year in Rhizo-Sus were *Bdellovibrionales bacterium* RBG 16 40 8 and *Humibacter ginsengisoli* bacteria, and *Oidiodendron maius*, *Oidiodendron truncatum*, *Pyrenochaetopsis leptospora*, and *Verticillium leptobactrum* fungi. The specific bacteria in Rhizo-Res were *Burkholderia* sp., *Rhizobiales bacterium* GAS113, and *Rudaea cellulosilytica* DSM 22992, and *Bifiguratus adelaidae* fungus.

### Analysis of Microbial Functions

**FIGURE 5:**
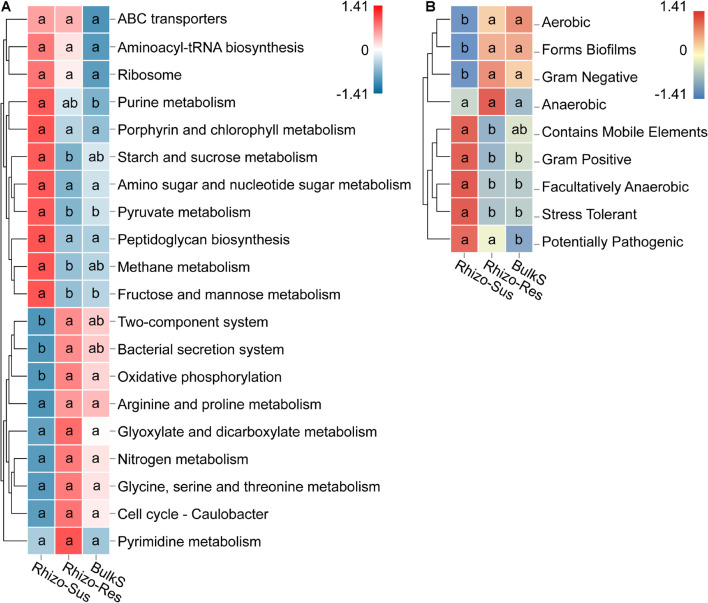
Heatmap of main functions based on relative abundance of bacterial KEGG prediction **(A)** using Tax4Fun and phenotype **(B)** using BugBase in rhizosphere of *C. oleifera* (Rhizo-Sus) and *C. yuhsienensis* (Rhizo-Res) and bulk soil (BulkS) samples. The abundance of functions was normalized by Z-score method. The connecting lines on the left side describe the clustering of each function. The closer the color to red, the more dominant the function is. The lowercase in each row of heatmap indicated the significant differences among Rhizo-Sus, Rhizo-Res, and BulkS (conducted using Wilcoxon rank sum test, *p* < 0.05), *N* = 12.

Microbial functions in the rhizosphere and bulk soil were defined based on KEGG and BugBase. We use the BugBase database term “phenotype,” which predicts organism-level microbiome phenotypes partly corresponding to microbial functional groups. The KEGG and phenotype heatmaps indicated that the functions and phenotypes of the bacteria in Rhizo-Res were like those in BulkS but different from those in Rhizo-Sus (Figure 5). The relative abundance of genes responsible for carbohydrate metabolism (starch and sucrose, pyruvate, and fructose and mannose) and for energy metabolism (methane metabolism) were higher in Rhizo-Sus than in Rhizo-Res (*p* < 0.05). The relative abundance of genes responsible for oxidative phosphorylation was higher in Rhizo-Res than in Rhizo-Sus. The microbiome phenotype heatmap based on BugBase showed that Gram-negative group in Rhizo-Res was higher than in Rhizo-Sus (*p* < 0.05). Gram-positive and stress-tolerant and facultatively anaerobic groups were higher in Rhizo-Sus than in Rhizo-Res (*p* < 0.05).

**FIGURE 6:**
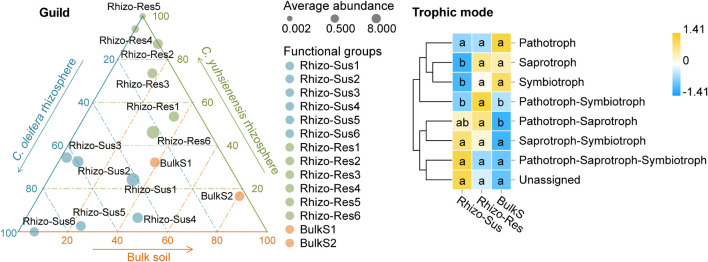
Distribution of fungal functional groups (guild) and heatmap of fungal trophic modes in rhizosphere and bulk soil. Ternary plot of guild plotted based *C. oleifera* rhizosphere (Rhizo-Sus), *C. yuhsienensis* rhizosphere (Rhizo-Res), and bulk soil (BulkS) specificity. The circle size, color, and position indicate the average abundance, guild, and affiliation with Rhizo-Sus, Rhizo-Res, and BulkS (detected by Kruskal–Wallis analysis, *p* < 0.05), respectively. Rhizo-Sus 1–6 are Undefined Saprotroph, Endophyte–Fungal Parasite–Plant Pathogen, Ericoid Mycorrhizal, Endophyte–Lichen Parasite–Undefined Saprotroph, Endomycorrhizal–Plant Pathogen–Undefined Saprotroph, and Animal Pathogen–Endophyte–Plant Saprotroph–Soil Saprotroph, respectively. Rhizo-Res 1–6 are Arbuscular Mycorrhizal, Undefined Saprotroph-Undefined Biotroph, Endophyte–Plant Pathogen, Lichenized, Animal Pathogen–Endophyte–Lichen Parasite–Plant Pathogen–Wood Saprotroph, and Unassigned, respectively. BulkS 1 and 2 are Animal Pathogen–Fungal Parasite–Undefined Saprotroph and Dung Saprotroph–Undefined Saprotroph, respectively. The abundance of functions was normalized by Z-score method. The connecting lines on the left side describe the clustering of each function. The closer the color to yellow, the more dominant the function is. Pathotroph = receiving nutrients by harming host cells; symbiotroph = receiving nutrients by exchanging resources with host cells; saprotroph = receiving nutrients by breaking down dead host cells. The lowercase in each row of heatmap indicated the significant differences among Rhizo-Sus, Rhizo-Res, and BulkS (conducted using Wilcoxon rank sum test, *p* < 0.05), *N* = 12.

The analysis of fungal functional groups (guilds) and trophic mode showed that endophytes, plant pathogens, and mycorrhizal guilds were present in both Rhizo-Sus and Rhizo-Res (Figure 6). The symbiotrophs and saprotrophs in Rhizo-Res were higher in Rhizo-Sus (*p* < 0.05), but pathotrophs were similar between Rhizo-Sus and Rhizo-Res (Figure 6).

### Effects of Physicochemical Properties on Microbial Communities

The relationships between microbial communities and physiochemical properties were not consistent among Rhizo-Sus, Rhizo-Res, and BulkS (Supplementary Tables 6, 7). Alkaline hydrolyzable nitrogen (AMN) and AK decreased (*p* < 0.01) with increasing bacterial alpha diversity in Rhizo-Sus and Rhizo-Res, respectively (Supplementary Table 6). Soil moisture had no effects on bacterial alpha diversity in Rhizo-Sus (*p* > 0.05). The effects of moisture on bacterial alpha diversity in Rhizo-Res was positive (*p* < 0.05), whereas it was negative in bulk soils (*p* < 0.05).

Available phosphorous increased with (*p* < 0.05) fungal richness in all soil conditions (Supplementary Table 7). Total nitrogen increased with fungal richness in Rhizo-Sus and BulkS and fungal diversity in Rhizo-Res (*p* < 0.05). C/P ratio increased (*p* < 0.05) with fungal diversity in Rhizo-Sus and Rhizo-Res but not in BulkS.

## Discussion

### Specific Microorganisms in Each *Camellia* spp. Rhizosphere

#### Specific Bacteria in Each *Camellia* spp. Rhizosphere and Their Functions

Despite partial overlap of bacterial communities in the rhizosphere of susceptible *C. oleifera*, resistant *C. yuhsienensis*, and bulk soil, some bacterial communities were specific and were distributed solely under one of the *Camellia* spp. (Figure 4 and Table 1). Most *B. epidermidis*, *Proteus mirabilis*, and *Rhodococcus erythropolis* were specific bacteria found in the rhizosphere of susceptible *C. oleifera* and are PGPR and biocontrol bacteria (Figure 3 and Table 1). *B. epidermidis* is a very important N_2_-fixing and P-solubilizing bacterium living in the rhizosphere (Karagoz et al., 2012). *B. epidermidis* can increase length and dry weight of canola roots by producing indole acetic acid (IAA) and 1-aminocyclopropane-1-carboxylic acid (ACC) deaminase and by fixing N_2_ (Siddikee et al., 2010). *P. mirabilis* is a PGPR by solubilizing P and K, fixing N_2_, and producing IAA and phytase in cabbage and *Foeniculum vulgare* rhizosphere (Motamedi et al., 2016; Dhiman et al., 2019). *R. erythropolis* protects plant well from Gram-negative soft-rot bacteria by degrading their *N*-acyl-homoserine lactone signaling molecules (Latour et al., 2013). However, some deleterious bacteria were observed in Rhizo-Sus such as *Gryllotalpicola* sp., a specific species in the rhizosphere of resistant *C. yuhsienensis*, commonly found in the gut of wood-feeding insects and is associated with pinewood nematode and pine wilt disease (Fang et al., 2015; Guo et al., 2020). Consequently, pathogenic insects are common in the rhizosphere of *C. oleifera*.

**TABLE 1:** Main known functions of bacteria specific for rhizosphere of *C. oleifera* (Rhizo-Sus) and *C. yuhsienensis* (Rhizo-Res).

Specific in	Microorganisms	RB	Functions	References
Rhizo-Sus	*Brevibacterium epidermidis*	0.08%	N_2_-fixing, P-solubilizing, producing IAA, increasing length and dry weight of plant roots	Karagoz et al., 2012
	*Rhodococcus erythropolis*	0.01%	Against plant soft rot pathogen	Latour et al., 2013
	*Proteus mirabilis*	0.002%	N_2_-fixing, P- and K-solubilizing, producing IAA and phytase	Motamedi et al., 2016; Dhiman et al., 2019
	
	*Gryllotalpicola* sp.	0.01%	Associated with wood-feeding insects and pine wilt disease	Fang et al., 2015; Guo et al., 2020
	
Rhizo-Res	*Acinetobacter lwoffii*	0.003%	P-solubilizing, producing IAA and exopolysaccharide	Das and Sarkar, 2018
	*Staphylococcus sciuri*	0.001%	Antagonism against strawberry anthracnose by producing volatile compounds	Alijani et al., 2019
	*Burkholderia* sp.	0.12%	N_2_-fixing, formatting nodulation in the root of legume plants (*Burkholderia tuberum*, *Burkholderia phymatum*)	Moulin et al., 2001; Vandamme et al., 2003
			P-solubilizing, antagonism against several pathogenic fungi (*Burkholderia cepacia*)	Zhao et al., 2014
			Reducing plant heavy metal toxicity (*Burkholderia* sp. J62, *Burkholderia* sp. SCMS54)	Jiang et al., 2008; Dourado et al., 2013
	
			Wilt pathogenic bacteria of carnation (*Burkholderia caryophylli*)	Ragupathi and Veeraraghavan, 2019
			Causing blight of rice (*Burkholderia glumae*)	Ham et al., 2011

*RB indicates the relative abundance of bacteria in the rhizosphere of C. oleifera or C. yuhsienensis.*

*Acinetobacter lwoffii* and *Staphylococcus sciuri*, the specific bacteria in Rhizo-Res, are PGPR and biocontrol bacteria. *A. lwoffii* can produce IAA and exopolysaccharide (EPS), which are capable of solubilizing P to promote mung bean growth (Das and Sarkar, 2018). *S. sciuri* is a biocontrol bacterium of strawberry anthracnose, as it produces volatile compounds (VOCs) that can suppress mycelial growth and conidial germination of *Colletotrichum nymphaeae* (Alijani et al., 2019). *Burkholderia* sp. has wide ecological niches. Some species in this genus are plant pathogens. For instance, *Burkholderia caryophylli* is the wilt pathogenic bacteria of carnation (Ragupathi and Veeraraghavan, 2019). *Burkholderia glumae* is one of the major pathogens of rice that causes blight (Ham et al., 2011). Some are reported as PGPR (Moulin et al., 2001; Vandamme et al., 2003) and have the ability to control plant pathogens (Coenye and Vandamme, 2003; Zhao et al., 2014) and reduce heavy metal toxicity, such as cadmium and palladium (Jiang et al., 2008; Dourado et al., 2013). These results indicate that the specific bacteria in rhizosphere of susceptible *C. oleifera* and resistant *C. yuhsienensis* have potential positive and negative influence on plants.

#### Specific Fungi in Each *Camellia* spp. Rhizosphere and Their Functions

The major known functions of specific fungi in the rhizosphere of susceptible *C. oleifera* and resistant *C. yuhsienensis* are presented in Table 2. Most specific fungi in Rhizo-Sus can increase plant growth and control pathogens. *O. maius* is an important ericoid mycorrhizal fungus in rhizosphere of *Rhododendron* spp. (Vohník et al., 2005). *O. maius* can increase *Rhododendron fortune* fresh and dry weight by upregulating nitrate transporters, ammonium transporter, glutamine synthetase, and glutamate synthase in plants to increase N uptake (Wei et al., 2016). *V. leptobactrum* protects plants from root knot nematodes by suppressing growth of eggs and second-stage juveniles (Regaieg et al., 2011; Hajji et al., 2017). *Candida intermedia* is able to control strawberry fruit rot by producing volatile organic compounds to suppress conidial germination and mycelial growth of *B. cinerea* (Huang et al., 2011). *C. intermedia* also has the ability to reduce anthracnose incidence in avocado fruits by restraining mycelia growth of *Colletotrichum gloeosporioides* (Campos-Martínez et al., 2016). *Chrysosporium pseudomerdarium* is an endophyte that can increase plant shoot length and chlorophyll content by producing gibberellins to promote plant growth (Hamayun et al., 2009; Waqas et al., 2014). *Lecanicillium fusisporum* is an important biocontrol endophytic fungus against wheat disease *Septoria tritici* blotch caused by *Zymoseptoria tritici* (Latz et al., 2020).

**TABLE 2:** Main known functions of fungi specific for rhizosphere of *C. oleifera* (Rhizo-Sus) and *C. yuhsienensis* (Rhizo-Res).

Specific in	Microorganisms	RB	Functions	References
Rhizo-Sus	*Verticillium leptobactrum*	1.83%	Suppressing growth of root knot nematodes’ eggs and second-stage juveniles	Regaieg et al., 2011; Hajji et al., 2017
	*Oidiodendron maius*	0.36%	Increasing plant N uptake	Wei et al., 2016
	*Candida intermedia*	0.28%	Producing volatile organic compounds to control fruit rot of strawberry, reducing anthracnose incidence in avocado fruits by restraining mycelia growth of *Colletotrichum gloeosporioides*	Huang et al., 2011; Campos-Martínez et al., 2016
	*Chrysosporium pseudomerdarium*	0.03%	Producing gibberellins to increase plant shoot length and chlorophyll content	Hamayun et al., 2009; Waqas et al., 2014
	*Lecanicillium fusisporum*	0.03%	Antagonism against wheat disease Septoria tritici blotch	Latz et al., 2020
	
	*Ustilaginoidea virens*	0.50%	Causing false smut on rice	Brooks et al., 2009
	*Cyphellophora sessilis*	0.01%	Causing Sooty blotch and flyspeck on apple	Batzer et al., 2015
	
Rhizo-Res	*Cenococcum geophilum*	0.09%	Producing melanin to improve plant resistant to water stress	Jany et al., 2003
	*Metarhizium anisopliae*	0.03%	Protecting wheat from *Sitophilus oryzae*	Batta, 2004
	*Monocillium bulbillosum*	0.01%	Parasitizing the eggs of *Heterodera filipjevi* to protect plant	Ashrafi et al., 2017
	*Veronaeopsis simplex*	0.01%	Suppressing Fusarium wilt disease	Khastini et al., 2012
	*Schizophyllum commune*	0.01%	Causing brown germ and seed rot of oil palm	Dikin et al., 2006

*RB indicates the relative abundance of bacteria in the rhizosphere of C. oleifera or C. yuhsienensis.*

Phytopathogens were also observed in Rhizo-Sus. *Cyphellophora sessilis* (Batzer et al., 2015) and *Ustilaginoidea virens* (Brooks et al., 2009) are common phytopathogens on apple and rice, respectively. *Fusarium concentricum* causes rot disease in several plants, such as pepper fruit rot and *Paris polyphylla* stem rot (Wang et al., 2013; Xiao et al., 2019). On the phylogenetic tree, *F. concentricum* is very close to *Fusarium proliferatum*, which is a root rot phytopathogen of oil tea (Li et al., 2008).

Most specific fungi in Rhizo-Res are plant-growth-promoting species. *Cenococcum geophilum* is an important ectomycorrhizal fungus that maintain the physiological integrity of beech roots facing drought stress (Jany et al., 2003). Melanin, produced by *C. geophilum*, improved plant resistant to water stress (Fernandez and Koide, 2013). *Metarhizium anisopliae* and *Monocillium bulbillosum* are important biocontrol fungi for insect pest. *M. anisopliae* conidia with oven ash, chalk powder, charcoal, and wheat flour resulted in 73–87% mortality of adult *Sitophilus oryzae* (Batta, 2004). *M. bulbillosum* protected plants from nematodes by parasitizing the eggs of *Heterodera filipjevi* (Ashrafi et al., 2017). *Veronaeopsis simplex* is a dark septate endophytic fungus with the ability to suppress *Fusarium* wilt disease in Chinese cabbage (Khastini et al., 2012). There was also a phytopathogen in Rhizo-Res, named *Schizophyllum commune*, that causes brown germ and seed rot of oil palm (Dikin et al., 2006).

In summary, the abundance of specific pathogenic microorganisms in the rhizosphere of resistant *C. yuhsienensis* was too low to have similar negative effects on plant growth and health compared to susceptible *C. oleifera* (Tables 1, 2).

### Microbial Community Association With *Camellia* spp. Growth and Health

Roots and rhizodeposition of *Camellia* species are important factors of bacterial and fungal abundance, diversity, structure, and function (Figures 1, 2, Tables 1, 2, Supplementary Figures 3, 4, and Supplementary Table 5; Zhang P. et al., 2020). Although the relationship between roots and rhizosphere microorganisms is mostly mutualistic (Qiang et al., 2012; Mendes et al., 2013), competitive relationships do exist in many cases, such as the competition for nitrogen (Hodge et al., 2000; Kuzyakov and Xu, 2013) especially under stress conditions (Xu et al., 2011). Bacteria compete with plants by assimilating and immobilizing P and N using organic carbon (Zhang et al., 2014), and this competition gets more severe due to the root exudates, including available carbon substrates for bacteria (Kuzyakov and Xu, 2013; Kuzyakov et al., 2019).

The BugBase prediction showed that the abundance of Gram-positive, facultatively anaerobic, and stress-tolerant bacteria were higher in Rhizo-Sus than in Rhizo-Res and BulkS, while Gram-negative bacteria had the opposite trend (*p* < 0.05) (Figure 5B). Gram-positive bacteria are generally considered as environmental stress-tolerant bacteria compared with Gram-negative bacteria (Schimel et al., 2007). Our study (Supplementary Table 2) agrees with previous studies (Liu et al., 2018), which indicated that growth of oil tea is limited by insufficient nutrients in the red soil area. The productivity of *C. oleifera* is higher than *C. yuhsienensis*, which means that *C. oleifera* has a greater ability to mine and absorb nutrients (Tan et al., 2011). The larger crown width, trunk diameter, and yield of *C. oleifera* also indicated that its nutrient utilization efficiency is higher than that of *C. yuhsienensis* (Supplementary Table 1). Finally, *C. oleifera* formed a microbial community of lower richness and diversity but of greater activity of carbohydrate metabolism. In contrast, more bacteria with oxidative phosphorylation and more Gram-negative bacteria indicated that the microbial communities acquired more high-quality substrates in the rhizosphere of *C. yuhsienensis*. More facultatively anaerobic bacteria implied a poorer water permeability condition in the soil under *C. oleifera* (Figure 5). Based on these results, we speculate that *C. oleifera* formed competitive relationships with the rhizosphere microbial communities, compared to the symbiotic interactions of *C. yuhsienensis* in the rhizosphere. These differences in relationships may be one of the most important factors for specific effects of microorganisms on oil tea growth and health.

The rhizosphere microbial diversity is an important factor in plant health and ecosystem function (Xue et al., 2020), excluding the negative effects of pathotrophs fungi plant health (Mendes et al., 2013; Nguyen et al., 2016). The bacterial and fungal diversity and the relative abundance of Acidobacteria were higher in *C. yuhsienensis* rhizosphere than in *C. oleifera* (Supplementary Table 5, Figure 1, and Supplementary Figure 3). Bacterial richness and diversity increase in soil if pathogens are suppressed. This was clearly shown in soils with suppressed *Fusarium* disease than in soils with serious *Fusarium* wilt disease (Shen et al., 2015). By studying single and mixed strains of four bacterial species antagonism on plant pathogens, Boer et al. (2007) concluded that (1) more bacteria in soil can lead to stronger competition with pathogens for resources and (2) interactions among bacteria increase antifungal activity. When biocontrol bacteria or fungi meet other microorganisms with similar functions, these microorganisms increased the production of antibiotics, such as 2,4-diacetylphloroglucinol (DAPG) (Lutz et al., 2004; Maurhofer et al., 2004). In other words, a microbial community of higher diversity has a higher potential to antagonize pathogens. Therefore, according to bacterial and fungal diversity, we state that the microbial community in *C. yuhsienensis* rhizosphere better protects the host from pathogens than the microbial community in *C. oleifera* rhizosphere (Supplementary Table 5).

## Conclusion

The dominant bacteria in the rhizosphere of oil tea (*Camellia* spp.) were Chloroflexi, Acidobacteria, Proteobacteria, Actinobacteria, and Planctomycetes, and the dominant fungi were Ascomycota and Basidiomycota. *Camellia* species plays a crucial role in microbial richness, diversity, and community structure of its rhizosphere. The rhizosphere of both *Camellia* spp. contains beneficial and deleterious microorganisms. Numbers and abundance of deleterious microorganisms were more in *C. oleifera* than that in *C. yuhsienensis*. The bacterial groups and functions in the rhizosphere of disease-resistant *C. yuhsienensis* were similar to those in bulk soil but much different from those in the rhizosphere of disease susceptible *C. oleifera*. More Gram-negative bacteria were in the rhizosphere of *C. yuhsienensis*, while more Gram-positive, facultatively anaerobic, and stress-tolerant bacteria were in the rhizosphere of *C. oleifera*. There was higher bacterial and fungal alpha diversity in the rhizosphere of *C. yuhsienensis* than *C. oleifera*. Our results indicate that the more abundant and diverse microbial community in *C. yuhsienensis* rhizosphere better protects the host from pathogens compared to those in *C. oleifera* rhizosphere.

## Data Availability Statement

The data presented in the study are deposited in the NCBI Sequence Read Archive (SRA) database, accession number: PRJNA742848.

## Author Contributions

JY and JL: conceptualization and validation. JL: methodology, formal analysis, data curation, and writing – original draft preparation. JL, CZ, XQ, and ZL: investigation. JL, SL, YK, HA, JY, and GN: writing, review, and editing. JL and YK: visualization. JY and GN: supervision. JY, JL, and YK: funding acquisition. All authors have read and agreed to the published version of the manuscript.

## Conflict of Interest

The authors declare that the research was conducted in the absence of any commercial or financial relationships that could be construed as a potential conflict of interest.

## Publisher’s Note

All claims expressed in this article are solely those of the authors and do not necessarily represent those of their affiliated organizations, or those of the publisher, the editors and the reviewers. Any product that may be evaluated in this article, or claim that may be made by its manufacturer, is not guaranteed or endorsed by the publisher.
