# Supplementary material for: Microbial Communities and Functions in the Rhizosphere of Disease-Resistant and Susceptible Camellia spp
Source: Front Microbiol. 2021 Oct 18;12:732905. doi: 10.3389/fmicb.2021.732905 (PMC8558623; doi:10.3389/fmicb.2021.732905)
Supplement: Supplementary file 1 [file Data_Sheet_1.docx]

**Supplementary Table 1**.The growth characters of *Camellia oleifera* and *Camelliayuhsienensis*.

| Species | Repeats | Tree Height  (cm) | Crown Width  (cm) | Ground Diameter  (cm) | Average fresh fruit yield  (kg/plant) | Average oil yield  (g/plant) | Anthracnose infection rate  (%) |
| --- | --- | --- | --- | --- | --- | --- | --- |
| *C. oleifera* | 1 | 272 | 288 | 15.0 | 15 to 20 | 825 to 1170 | 2 to 80 |
|  | 2 | 401 | 339 | 14.0 |  |  |  |
|  | 3 | 304 | 256 | 12.1 |  |  |  |
|  | 4 | 274 | 218 | 10.7 |  |  |  |
|  | 5 | 282 | 237 | 10.5 |  |  |  |
|  | 6 | 283 | 263 | 11.3 |  |  |  |
|  | 7 | 313 | 335 | 14.3 |  |  |  |
|  | 8 | 305 | 245 | 12.4 |  |  |  |
|  | 9 | 330 | 282 | 16.4 |  |  |  |
|  | 10 | 312 | 322 | 11.5 |  |  |  |
| *C. yuhsienensis* | 1 | 305 | 136 | 7.5 | 1.4 to 2 | 105 to 170 | 0 to 10 |
|  | 2 | 321 | 87 | 7.7 |  |  |  |
|  | 3 | 326 | 88 | 6.7 |  |  |  |
|  | 4 | 340 | 134 | 7.2 |  |  |  |
|  | 5 | 270 | 105 | 6.8 |  |  |  |
|  | 6 | 313 | 80 | 8.0 |  |  |  |
|  | 7 | 261 | 90 | 6.7 |  |  |  |
|  | 8 | 216 | 83 | 3.8 |  |  |  |
|  | 9 | 276 | 103 | 5.7 |  |  |  |
|  | 10 | 310 | 126 | 6.7 |  |  |  |

**Supplementary Table 2**. Soil physico-chemical properties.

| Environ-factors | Rhizo-Sus | | | | Rhizo-Res | | | | BulkS | | | |
| --- | --- | --- | --- | --- | --- | --- | --- | --- | --- | --- | --- | --- |
|  | Spring | Summer | Autumn | Winter | Spring | Summer | Autumn | Winter | Spring | Summer | Autumn | Winter |
| AP (mg/kg) | 9.5±0.1b | 12.8±0.4a | 3.3±0.0e | 6.5±0.2c | 9.5±0.3b | 5.4±1.0d | 1.3±0.2f | 2.6±0.0e | 9.7±0.2b | 6.3±0.8cd | 0.5±0.2g | 3.2±0.2e |
| TP (mg/kg) | 476±6bc | 563±30a | 581±13a | 448±27cd | 440±15cde | 478±14bc | 510±8b | 415±9de | 399±7e | 465±23c | 354±32f | 348±33f |
| AK (mg/kg) | 143±10c | 169±7a | 106±2e | 114±1e | 130±3d | 159±6ab | 94±2f | 126±1d | 113±9e | 158±8ab | 112±3e | 153±1bc |
| TK (mg/kg) | 342±12cd | 331±10cd | 381±1a | 353±6bc | 335±19cd | 342±13cd | 374±3ab | 332±2cd | 367±13ab | 338±19cd | 381±4a | 329±7d |
| AMN (mg/kg) | 44.7±8.7ef | 60.5±5.2cd | 80.7±4.6b | 107.9±11.6a | 29.6±1.7g | 61.3±15.0cd | 71.0±6.2bc | 59.2±5.6cde | 76.8±0.6b | 32.2±0.7fg | 82.2±4.5b | 55.8±4.2de |
| NN (mg/kg) | 53.0±3.5bc | 18.4±1.7e | 15.5±0.4e | 53.0±7.1bc | 35.0±2.5d | 19.1±1.1e | 38.6±2.2d | 57.9±10.2b | 38.5±2.6d | 69.6±12.6a | 20.6±1.6e | 42.2±4.3cd |
| AHN (mg/kg) | 104±2cd | 85±3e | 123±6b | 101±5d | 144±8a | 85±8e | 106±9cd | 116±3bc | 103±13cd | 101±5d | 76±7e | 149±4a |
| TN (mg/kg) | 1076±76ab | 1003±44bcd | 901±61ef | 828±3fg | 1150±9a | 1023±55bc | 945±56cde | 936±5de | 905±11ef | 844±25fg | 802±19g | 928±23de |
| TOC (g/kg) | 13.5±1.8ab | 11.8±1.0bc | 11.5±0.2bcd | 11.2±1.1cd | 14.7±0.4a | 12.0±0.7bc | 11.9±0.3bc | 12.8±2.0abc | 11.1±0.1cd | 12.0±0.9bc | 9.7±0.7d | 13.0±1.0abc |
| pH | 4.0±0.0b | 4.0±0.0b | 4.0±0.2b | 3.9±0.0b | 4.1±0.0ab | 4.4±0.0a | 4.2±0.3ab | 4.4±0.0a | 4.2±0.1ab | 4.4±0.0a | 4.3±0.0a | 4.3±0.4a |
| SWC | 0.3±0.0e | 0.2±0.0g | 0.3±0.0de | 0.3±0.0d | 0.3±0.0bc | 0.2±0.0f | 0.3±0.0de | 0.3±0.0b | 0.3±0.0cd | 0.2±0.0fg | 0.3±0.0de | 0.4±0.0a |
| C/N | 12.5±0.8ab | 11.8±0.7b | 12.9±1.1ab | 13.5±1.3ab | 12.7±0.3ab | 11.8±1.3b | 12.6±1.0ab | 13.7±2.2ab | 12.3±0.1ab | 14.3±1.4a | 12.1±0.6ab | 14.0±0.8ab |
| C/P | 28.4±4.0bcd | 21±1.3ef | 19.9±0.8f | 25.2±4.0def | 33.3±0.5ab | 25.1±0.8def | 23.3±0.3def | 31.0±5.5bc | 27.9±0.5bcd | 26±2.9cde | 27.7±3.3bcd | 37.5±1.9a |
| N/P | 2.3±0.2b | 1.8±0.1cd | 1.6±0.1d | 1.9±0.1c | 2.6±0.1a | 2.1±0.2b | 1.9±0.1c | 2.3±0.0b | 2.3±0.0b | 1.8±0.1cd | 2.3±0.2b | 2.7±0.2a |

Rhizo-Sus, Rhizo-Res and BulkS indicates *C. oleifera* rhizosphere, *C.yuhsienensis* rhizosphere and bulk soil, respectively. AP, available phosphorous; TP, total phosphorous; AK, available potassium; TK, total potassium; AMN, ammonium nitrogen; NN, nitrate nitrogen; AHN, alkaline hydrolyzable nitrogen; TN, total nitrogen; TOC, total organic carbon; SWC, soil water content; Tem, soil monthly mean temperature; C/N, ratio of total carbon to total nitrogen; C/P, ratio of total carbon to total phosphorous; N/P, ration of total nitrogen to total phosphorous. Numbers before and behind “±” are mean value (N=3) and SD, respectively. Lowercases behind numbers indicate significant differences (*p*<0.05).

**Supplementary Table 3**.Quantitative statistics of bacterial OTUs and Tags of each sample.

| Samples | Total Tags | Unique Tags | Taxon Tags | Unclassified Tags | Singleton Tags | OTUs |
| --- | --- | --- | --- | --- | --- | --- |
| Sp-Rhizo-Sus-1 | 131241 | 98800 | 109443 | 0 | 21798 | 1971 |
| Sp-Rhizo-Sus-2 | 118723 | 99553 | 84557 | 0 | 34166 | 2256 |
| Sp-Rhizo-Sus-3 | 154166 | 126224 | 110453 | 0 | 43713 | 2355 |
| Su-Rhizo-Sus-1 | 96472 | 68268 | 83457 | 0 | 13015 | 1563 |
| Su-Rhizo-Sus-2 | 94828 | 66852 | 80300 | 0 | 14528 | 1680 |
| Su-Rhizo-Sus-3 | 99047 | 69421 | 88114 | 0 | 10933 | 1558 |
| Au-Rhizo-Sus-1 | 117941 | 88942 | 90686 | 0 | 27255 | 1756 |
| Au-Rhizo-Sus-2 | 105561 | 63900 | 95639 | 0 | 9922 | 1318 |
| Au-Rhizo-Sus-3 | 115527 | 81007 | 93091 | 0 | 22436 | 1732 |
| Wi-Rhizo-Sus-1 | 120476 | 51587 | 116698 | 0 | 3778 | 740 |
| Wi-Rhizo-Sus-2 | 142432 | 69357 | 136017 | 0 | 6415 | 1005 |
| Wi-Rhizo-Sus-3 | 134866 | 60886 | 128791 | 0 | 6075 | 851 |
| Sp-Rhizo-Res-1 | 155371 | 129372 | 106782 | 0 | 48589 | 2408 |
| Sp-Rhizo-Res-2 | 135996 | 113976 | 94551 | 0 | 41445 | 2271 |
| Sp-Rhizo-Res-3 | 136625 | 114517 | 94121 | 0 | 42504 | 2347 |
| Su-Rhizo-Res-1 | 95998 | 65561 | 87963 | 0 | 8035 | 1366 |
| Su-Rhizo-Res-2 | 88087 | 59731 | 80371 | 0 | 7716 | 1317 |
| Su-Rhizo-Res-3 | 89003 | 54577 | 82225 | 0 | 6778 | 1101 |
| Au-Rhizo-Res-1 | 143142 | 122789 | 91269 | 0 | 51873 | 2370 |
| Au-Rhizo-Res-2 | 143884 | 123683 | 90297 | 0 | 53587 | 2396 |
| Au-Rhizo-Res-3 | 133713 | 113910 | 84763 | 0 | 48950 | 2426 |
| Wi-Rhizo-Res-1 | 139877 | 86517 | 132831 | 0 | 7046 | 1757 |
| Wi-Rhizo-Res-2 | 162967 | 98341 | 152097 | 0 | 10870 | 1881 |
| Wi-Rhizo-Res-3 | 210543 | 125029 | 196093 | 0 | 14450 | 1849 |
| Sp-BulkS-1 | 144138 | 108871 | 120561 | 0 | 23577 | 2013 |
| Sp-BulkS-2 | 117572 | 88053 | 98960 | 0 | 18612 | 2032 |
| Sp-BulkS-3 | 143436 | 111717 | 112117 | 0 | 31319 | 2194 |
| Su-BulkS-1 | 88735 | 73423 | 70400 | 0 | 18335 | 2001 |
| Su-BulkS-2 | 88366 | 71425 | 70062 | 0 | 18304 | 1947 |
| Su-BulkS-3 | 92053 | 74386 | 76200 | 0 | 15853 | 2052 |
| Au-BulkS-1 | 127676 | 100006 | 99926 | 0 | 27750 | 2064 |
| Au-BulkS-2 | 125204 | 102535 | 90288 | 0 | 34916 | 2247 |
| Au-BulkS-3 | 130349 | 106842 | 91726 | 0 | 38623 | 2180 |
| Wi-BulkS-1 | 191766 | 114931 | 177438 | 0 | 14328 | 1957 |
| Wi-BulkS-2 | 168986 | 104176 | 153046 | 0 | 15940 | 1887 |
| Wi-BulkS-3 | 183533 | 109688 | 171657 | 0 | 11876 | 1906 |
| Average | 128037 | 87805 | 106953 | 0 | 21083 | 1720 |

Sp, Su, Au and Wi indicates spring, summer, autumn and winter, respectively. Rhizo-Sus, Rhizo-Res and BulkS indicates rhizosphere soil of *C. oleifera*, rhizosphere soil of*C.yuhsienensis* and bulk soil sample, respectively. Number behind the “-“ indicates the replicate.

**Supplementary Table 4**.Quantitative statistics of fungal OTUs and Tags of each sample.

| Samples | Total Tags | Unique Tags | Taxon Tags | Unclassified Tags | Singleton Tags | OTUs |
| --- | --- | --- | --- | --- | --- | --- |
| Sp-Rhizo-Sus-1 | 228127 | 40282 | 225930 | 721 | 1476 | 866 |
| Sp-Rhizo-Sus-2 | 222744 | 48590 | 216587 | 1939 | 4218 | 1014 |
| Sp-Rhizo-Sus-3 | 194699 | 47502 | 188911 | 2526 | 3262 | 1058 |
| Su-Rhizo-Sus-1 | 93578 | 17931 | 91417 | 57 | 2104 | 621 |
| Su-Rhizo-Sus-2 | 87516 | 18494 | 85338 | 33 | 2145 | 728 |
| Su-Rhizo-Sus-3 | 92856 | 17628 | 90829 | 67 | 1960 | 674 |
| Au-Rhizo-Sus-1 | 105348 | 18689 | 103790 | 152 | 1406 | 447 |
| Au-Rhizo-Sus-2 | 134792 | 19949 | 132929 | 344 | 1519 | 516 |
| Au-Rhizo-Sus-3 | 98929 | 17427 | 96883 | 35 | 2011 | 558 |
| Wi-Rhizo-Sus-1 | 180932 | 22476 | 180679 | 3 | 250 | 396 |
| Wi-Rhizo-Sus-2 | 229233 | 44071 | 227375 | 684 | 1174 | 612 |
| Wi-Rhizo-Sus-3 | 221567 | 40344 | 217698 | 3477 | 392 | 393 |
| Sp-Rhizo-Res-1 | 210318 | 48922 | 206666 | 582 | 3070 | 1023 |
| Sp-Rhizo-Res-2 | 198089 | 46360 | 193555 | 689 | 3845 | 982 |
| Sp-Rhizo-Res-3 | 193572 | 49665 | 189871 | 923 | 2778 | 973 |
| Su-Rhizo-Res-1 | 87093 | 18893 | 84057 | 1663 | 1373 | 655 |
| Su-Rhizo-Res-2 | 96829 | 18889 | 92720 | 3318 | 791 | 569 |
| Su-Rhizo-Res-3 | 94149 | 18690 | 92786 | 563 | 800 | 594 |
| Au-Rhizo-Res-1 | 135054 | 35128 | 130493 | 1274 | 3287 | 714 |
| Au-Rhizo-Res-2 | 138696 | 37562 | 133061 | 1201 | 4434 | 785 |
| Au-Rhizo-Res-3 | 115729 | 31870 | 111247 | 916 | 3566 | 757 |
| Wi-Rhizo-Res-1 | 190313 | 39453 | 188027 | 912 | 1374 | 805 |
| Wi-Rhizo-Res-2 | 197121 | 30849 | 196798 | 32 | 291 | 510 |
| Wi-Rhizo-Res-3 | 173947 | 31567 | 172232 | 791 | 924 | 689 |
| Sp-BulkS-1 | 192724 | 39542 | 188488 | 2097 | 2139 | 737 |
| Sp-BulkS-2 | 188140 | 48967 | 185944 | 57 | 2139 | 705 |
| Sp-BulkS-3 | 160943 | 40504 | 156093 | 1057 | 3793 | 863 |
| Su-BulkS-1 | 99428 | 26508 | 95689 | 606 | 3133 | 753 |
| Su-BulkS-2 | 91348 | 22089 | 88158 | 248 | 2942 | 703 |
| Su-BulkS-3 | 88450 | 23203 | 85318 | 151 | 2981 | 685 |
| Au-BulkS-1 | 156310 | 22359 | 155165 | 528 | 617 | 480 |
| Au-BulkS-2 | 181931 | 27905 | 179024 | 350 | 2557 | 613 |
| Au-BulkS-3 | 137237 | 25677 | 133998 | 907 | 2332 | 610 |
| Wi-BulkS-1 | 168266 | 36162 | 165662 | 1125 | 1479 | 919 |
| Wi-BulkS-2 | 212898 | 43871 | 209357 | 482 | 3059 | 1004 |
| Wi-BulkS-3 | 217019 | 39914 | 214015 | 822 | 2182 | 933 |
| Average | 154246 | 29819 | 151244 | 975 | 2025 | 677 |

Sp, Su, Au and Wi indicates spring, summer, autumn and winter, respectively. Rhizo-Sus, Rhizo-Res and BulkS indicates rhizosphere soil of *C. oleifera*, rhizosphere soil of*C.yuhsienensis* and bulk soil sample, respectively. Number behind the “-“ indicates the replicate.

**Supplementary Table 5.** Alpha diversity (Sobs, Chao 1 and Shannon index) of bacteria and fungi in bulk and rhizosphere soil under *C. oleifera* and *C.yuhsienensis*.

| Kingdom | Sample | Sobs | Chao 1 | Shannon |
| --- | --- | --- | --- | --- |
| Bacteria | Sp-Rhizo-Sus | 2035±194bc | 2517±220bc | 8.52±0.02a |
|  | Su-Rhizo-Sus | 1544±74d | 1898±63e | 7.71±0.33de |
|  | Au-Rhizo-Sus | 1502±247d | 1895±204e | 7.84±0.21cd |
|  | Wi-Rhizo-Sus | 723±92f | 1142±80g | 7.01±0.34f |
|  | Sp-Rhizo-Res | 2170±44ab | 2656±35b | 8.46±0.04a |
|  | Su-Rhizo-Res | 1217±143e | 1545±99f | 7.48±0.30e |
|  | Au-Rhizo-Res | 2259±53a | 2871±116a | 8.33±0.07ab |
|  | Wi-Rhizo-Res | 1579±70d | 1868±107e | 8.29±0.03ab |
|  | Sp-BulkS | 1901±98c | 2275±148d | 8.26±0.14ab |
|  | Su-BulkS | 1989±38bc | 2346±23cd | 8.49±0.32a |
|  | Au-BulkS | 2048±99bc | 2433±154cd | 8.08±0.05bc |
|  | Wi-BulkS | 1622±8d | 1949±46e | 8.04±0.12bcd |
| Fungi | Sp-Rhizo-Sus | 455±95a | 658±80a | 5.75±0.64a |
|  | Su-Rhizo-Sus | 337±25bcd | 510±65cde | 2.84±0.29c |
|  | Au-Rhizo-Sus | 240±47de | 353±33fg | 2.68±0.40c |
|  | Wi-Rhizo-Sus | 172±42e | 296±88g | 4.33±1.98b |
|  | Sp-Rhizo-Res | 481±18a | 634±13ab | 5.92±0.28a |
|  | Su-Rhizo-Res | 303±27cd | 491±49cde | 5.02±0.14ab |
|  | Au-Rhizo-Res | 413±23ab | 537±64bcd | 5.18±0.18ab |
|  | Wi-Rhizo-Res | 278±116de | 439±47def | 5.29±0.62ab |
|  | Sp-BulkS | 436±131ab | 651±156ab | 5.59±0.55a |
|  | Su-BulkS | 406±19abc | 573±42abc | 4.94±0.62ab |
|  | Au-BulkS | 277±55de | 390±63efg | 4.87±0.44ab |
|  | Wi-BulkS | 424±29ab | 596±55abc | 5.40±0.33ab |

Sp, Su, Au and Wi indicates spring, summer, autumn and winter, respectively. Rhizo-Sus, Rhizo-Res and BulkS indicates *C. oleifera* rhizosphere, *C.yuhsienensis* rhizosphere and Non-rhizosphere soil sample, respectively. Numbers before and behind “±” are mean values (N=3) and SD, respectively. Lowercases behind numbers indicate significant differences (*p*<0.05)

**Supplementary Table 6**.Correlations between bacterial alpha-diversity and environmental factors based on Pearson correlation coefficients with two-tail test in in rhizosphere of *C. oleifera* (Rhizo-Sus) and *C.yuhsienensis* (Rhizo-Res) and bulk soil (BulkS).

| Environmental factors | Rhizo-Sus | | |  | Rhizo-Res | | |  | BulkS | | |
| --- | --- | --- | --- | --- | --- | --- | --- | --- | --- | --- | --- |
|  | Sobs | Chao 1 | Shannon |  | Sobs | Chao 1 | Shannon |  | Sobs | Chao 1 | Shannon |
| AP | 0.323 | 0.300 | 0.193 |  | 0.041 | 0.013 | -0.009 |  | -0.014 | -0.006 | 0.503 |
| TP | 0.276 | 0.209 | 0.162 |  | 0.245 | 0.344 | -0.208 |  | 0.398 | 0.374 | 0.676^*^ |
| AK | 0.429 | 0.396 | 0.310 |  | -0.798^**^ | -0.807^**^ | -0.713^**^ |  | -0.372 | -0.375 | 0.233 |
| TK | -0.242 | -0.233 | -0.160 |  | 0.425 | 0.458 | 0.073 |  | 0.646^*^ | 0.694^*^ | -0.099 |
| AMN | -0.841^**^ | -0.831^**^ | -0.791^**^ |  | -0.197 | -0.126 | -0.252 |  | 0.128 | 0.168 | -0.512 |
| NN | -0.140 | -0.058 | -0.045 |  | 0.256 | 0.175 | 0.604^*^ |  | -0.098 | -0.123 | 0.384 |
| AHN | 0.123 | 0.137 | 0.151 |  | 0.663^*^ | 0.575 | 0.823^**^ |  | -0.801^**^ | -0.757^**^ | -0.165 |
| TN | 0.684^*^ | 0.695^*^ | 0.657^*^ |  | 0.143 | 0.144 | -0.020 |  | -0.776^**^ | -0.733^**^ | -0.105 |
| TOC | 0.413 | 0.433 | 0.627^*^ |  | 0.285 | 0.251 | 0.441 |  | -0.649^*^ | -0.655^*^ | -0.026 |
| pH | 0.036 | 0.052 | -0.039 |  | -0.504 | -0.502 | -0.292 |  | -0.015 | -0.104 | 0.004 |
| SMC | -0.353 | -0.290 | -0.133 |  | 0.600^*^ | 0.516 | 0.884^**^ |  | -0.655^*^ | -0.591^*^ | -0.678^*^ |
| Tem | 0.605^*^ | 0.540 | 0.449 |  | -0.186 | -0.090 | -.641^*^ |  | 0.824^**^ | 0.765^**^ | 0.628^*^ |
| C/N | -0.315 | -0.305 | -0.004 |  | 0.160 | 0.128 | 0.447 |  | -0.310 | -0.343 | 0.061 |
| C/P | 0.090 | 0.143 | 0.318 |  | 0.100 | 0.032 | 0.423 |  | -0.816^**^ | -0.796^**^ | -0.550 |
| N/P | 0.323 | 0.381 | 0.404 |  | -0.010 | -0.067 | 0.145 |  | -0.697^*^ | -0.654^*^ | -0.644^*^ |

“*” and “**” indicate the significant correlations at 0.05 and 0.01 level, respectively. AP, available phosphorous; TP, total phosphorous; AK, available potassium; TK, total potassium; AMN, ammonium nitrogen; NN, nitrate nitrogen; AHN, alkaline hydrolyzable nitrogen; TN, total nitrogen; TOC, total organic carbon; SWC, soil water content; Tem, soil monthly mean temperature; C/N, ratio of total carbon to total nitrogen; C/P, ratio of total carbon to total phosphorous; N/P, ration of total nitrogen to total phosphorous.N=12.

**Supplementary Table 7**.Correlations between fungal alpha-diversity and environmental factors based on Pearson correlation coefficients with two-tail test in in rhizosphere of *C. oleifera* (Rhizo-Sus) and *C.yuhsienensis* (Rhizo-Res) and bulk soil (BulkS).

| Environmental factors | Rhizo-Sus | | |  | Rhizo-Res | | |  | BulkS | | |
| --- | --- | --- | --- | --- | --- | --- | --- | --- | --- | --- | --- |
|  | Sobs | Chao 1 | Shannon |  | Sobs | Chao 1 | Shannon |  | Sobs | Chao 1 | Shannon |
| AP | 0.541 | 0.596^*^ | 0.122 |  | 0.415 | 0.583^*^ | 0.575 |  | 0.567 | 0.661^*^ | 0.418 |
| TP | 0.011 | -0.038 | -0.771^**^ |  | 0.208 | 0.190 | -0.333 |  | 0.290 | 0.312 | -0.237 |
| AK | 0.628^*^ | 0.662^*^ | 0.052 |  | -0.350 | -0.139 | -0.069 |  | 0.345 | 0.287 | -0.002 |
| TK | -0.578^*^ | -0.595^*^ | -0.375 |  | 0.197 | 0.066 | -0.124 |  | -0.334 | -0.320 | 0.106 |
| AMN | -0.816^**^ | -0.807^**^ | -0.282 |  | -0.459 | -0.475 | -0.759^**^ |  | -0.333 | -0.279 | 0.208 |
| NN | 0.131 | 0.172 | 0.641^*^ |  | -0.313 | -0.334 | -0.069 |  | 0.423 | 0.459 | -0.198 |
| AHN | -0.161 | -0.297 | -0.010 |  | 0.579^*^ | 0.620^*^ | 0.657^*^ |  | 0.490 | 0.463 | 0.407 |
| TN | 0.670^*^ | 0.730^**^ | 0.226 |  | 0.466 | 0.566 | 0.582^*^ |  | 0.634^*^ | 0.679^*^ | 0.561 |
| TOC | 0.366 | 0.514 | 0.451 |  | 0.565 | 0.538 | 0.663^*^ |  | 0.488 | 0.519 | -0.038 |
| pH | 0.211 | 0.201 | -0.049 |  | -0.495 | -0.428 | -0.419 |  | -0.200 | -0.115 | -0.377 |
| SMC | -0.423 | -0.409 | 0.301 |  | 0.315 | 0.179 | 0.497 |  | 0.053 | 0.079 | 0.306 |
| Tem | 0.454 | 0.438 | -0.408 |  | 0.142 | 0.243 | -0.239 |  | -0.181 | -0.184 | -0.372 |
| C/N | -0.360 | -0.251 | 0.325 |  | 0.205 | 0.087 | 0.212 |  | 0.244 | 0.255 | -0.395 |
| C/P | 0.221 | 0.358 | 0.782^**^ |  | 0.312 | 0.283 | 0.619^*^ |  | 0.170 | 0.165 | 0.190 |
| N/P | 0.492 | 0.578^*^ | 0.745^**^ |  | 0.230 | 0.307 | 0.602^*^ |  | 0.018 | 0.007 | 0.441 |

“*” and “**” indicate the significant correlations at 0.05 and 0.01 level, respectively. AP, available phosphorous; TP, total phosphorous; AK, available potassium; TK, total potassium; AMN, ammonium nitrogen; NN, nitrate nitrogen; AHN, alkaline hydrolyzable nitrogen; TN, total nitrogen; TOC, total organic carbon; SWC, soil water content; Tem, soil monthly mean temperature; C/N, ratio of total carbon to total nitrogen; C/P, ratio of total carbon to total phosphorous; N/P, ration of total nitrogen to total phosphorous.N=12.


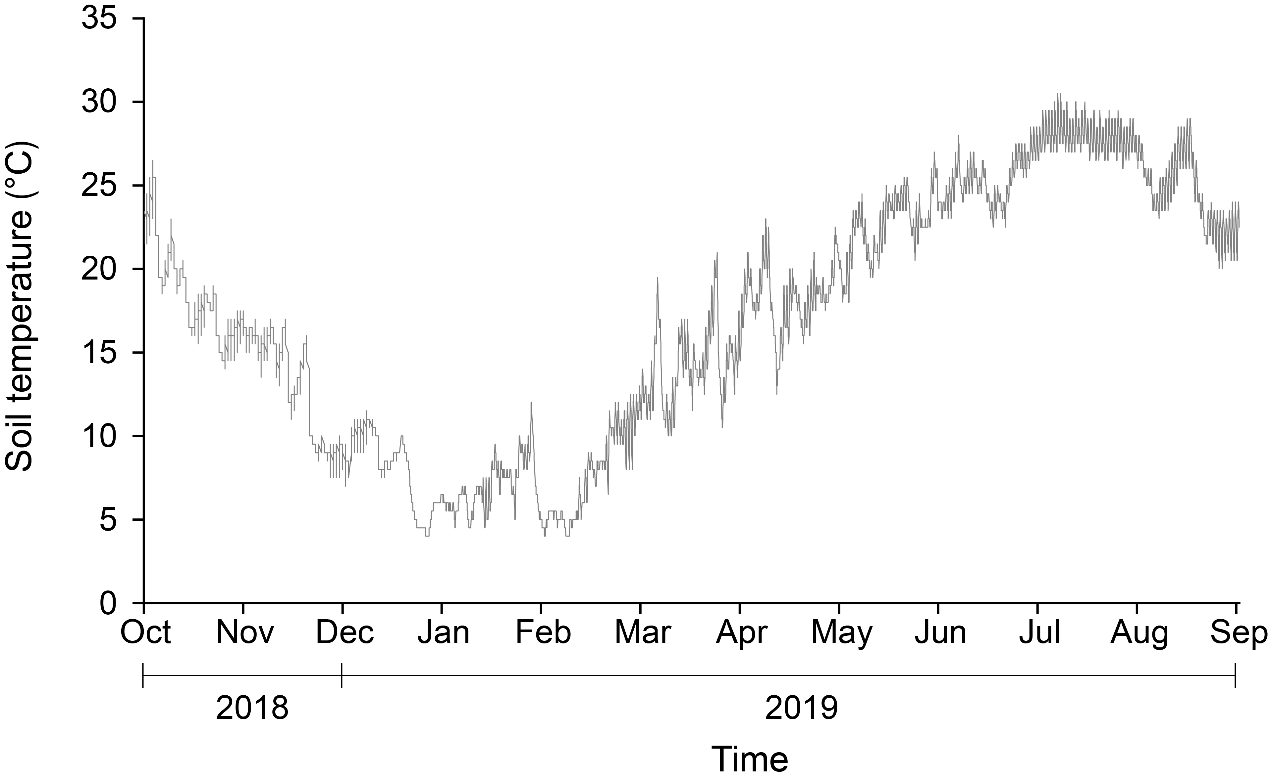


**Supplementary Figure 1.**Dynamics of soil temperature in the forest at 0–20 cm depth.


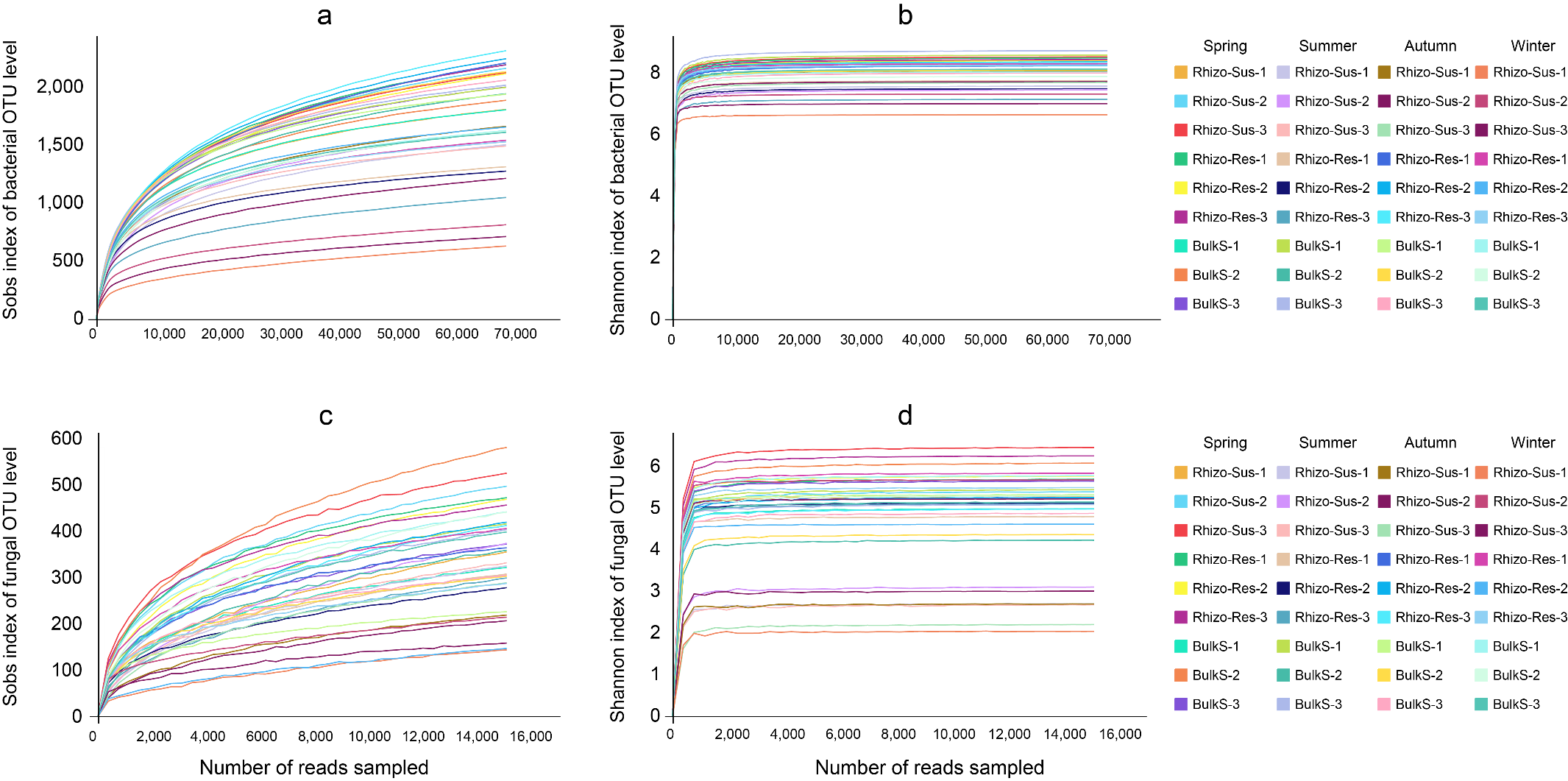


**Supplementary Figure 2**. Dilution curves of Sobs index of bacteria (a) and fungus (c) and Shannon index of bacteria (b) and fungus (d). Rhizo-Sus, Rhizo-Res and BulkS indicates *C. oleifera*rhizosphere, *C.yuhsienensis*rhizosphereand bulk soil sample, respectively.


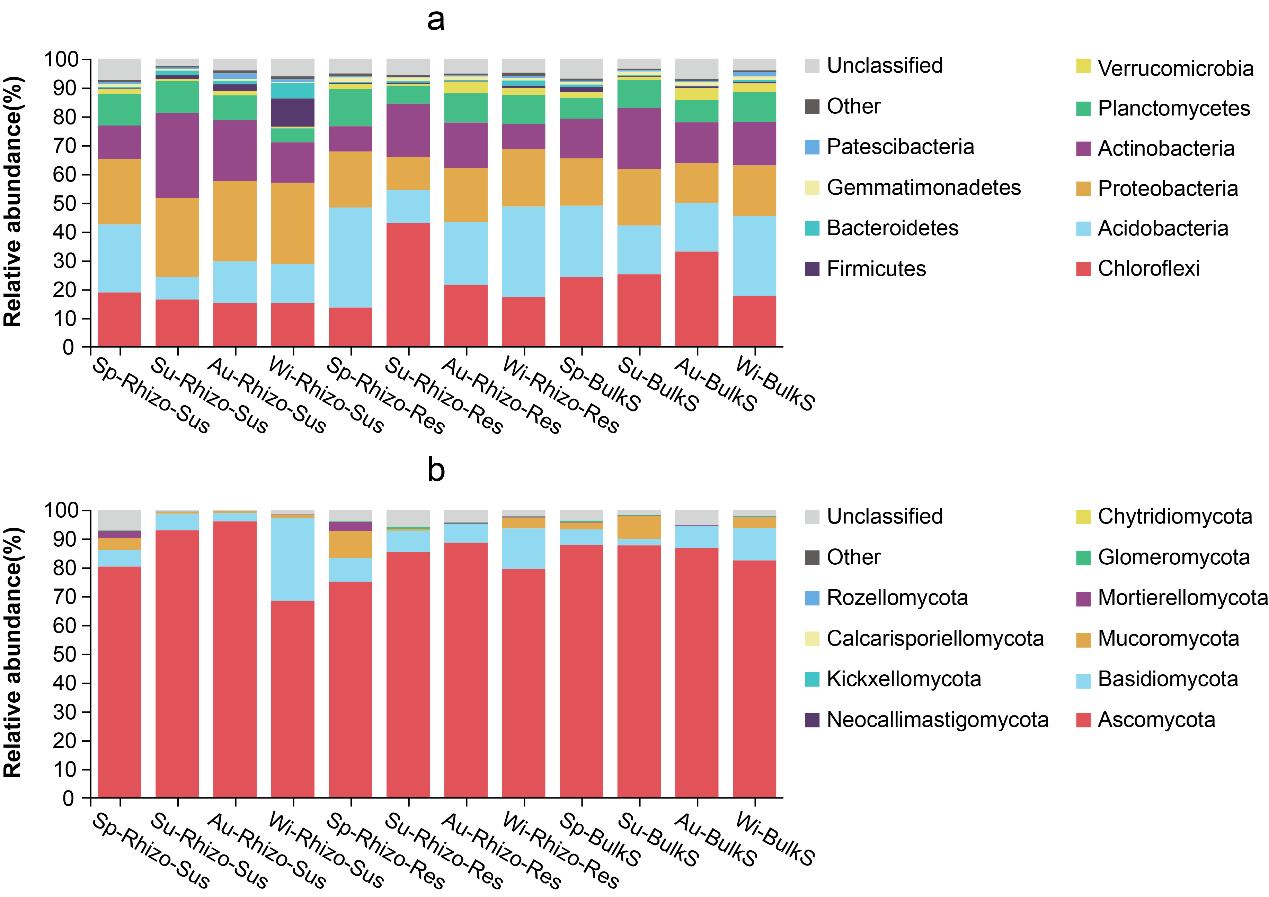


**Supplementary Figure 3**. Relative abundance of bacteria (a) and fungi (b) at phylum level. Sp, Su, Au and Wi indicates spring, summer, autumn and winter, respectively. Rhizo-Sus, Rhizo-Res and BulkS indicates *C. oleifera* rhizosphere, *C.yuhsienensis* rhizosphere and bulk soil sample, respectively. N=3.


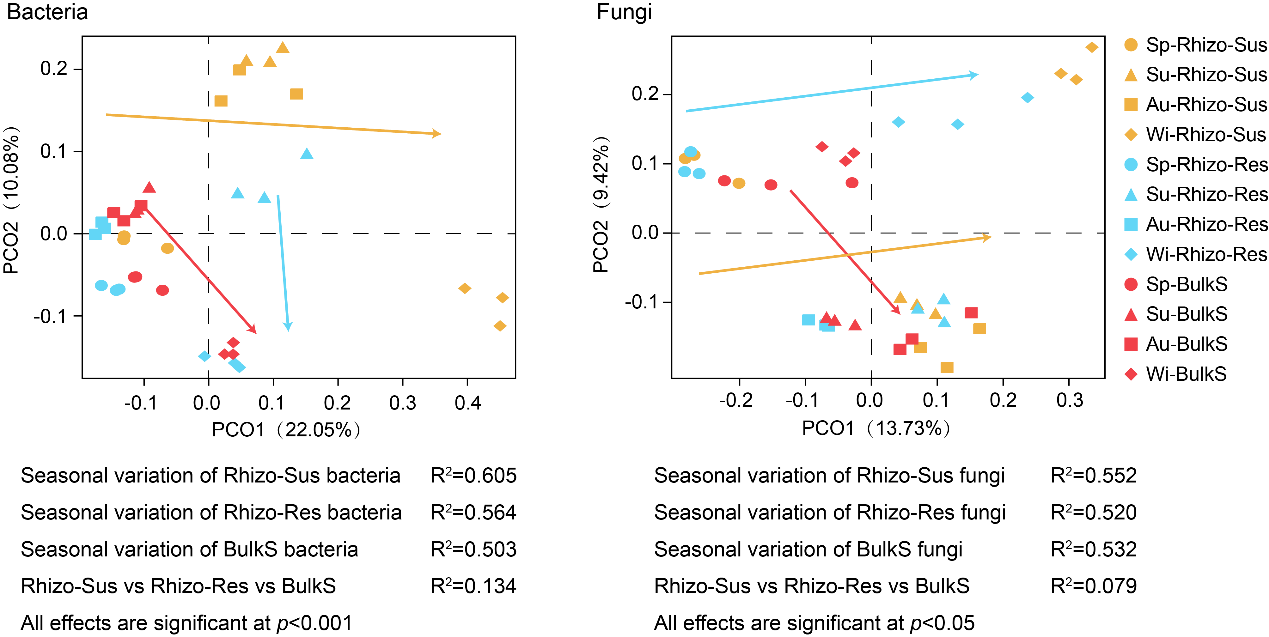


**Supplementary Figure 4**.Principal Co-ordinate Analysis (PCoA) of bacterialand fungal communitiesbased on unweighted unifrac distance.Sp, Su, Au and Wi indicates spring, summer, autumn and winter, respectively.Rhizo-Sus, Rhizo-Res and BulkS indicates *C. oleifera* rhizosphere, *C.yuhsienensis* rhizosphere and bulk soil, respectively. Values of R^2^ and *p* were calculated using Permutational MANOVA (Permanova). N=3.The yellow, azure and red arrows indicate the variation of bacterial (or fungal) communities in Rhizo-Sus, Rhizo-Res and BulkS along seasons, respectively.
